# Molecular Detection of Enteric Protozoa in Cattle from North Portugal

**DOI:** 10.3390/pathogens15090937

**Published:** 2026-09-04

**Authors:** Sara Gomes-Gonçalves, Ricardo J. Figueiredo, Beatriz Oliveira Alcobia, Luís Cardoso, Ana Cláudia Coelho, João R. Mesquita

**Affiliations:** 1 School of Medicine and Biomedical Sciences (ICBAS), Universidade do Porto (UP), 4050-313 Porto, Portugal; 2 Department of Veterinary Sciences, Animal and Veterinary Research Centre (CECAV), Universidade de Trás-os-Montes e Alto Douro (UTAD), Quinta de Prados, 5000-801 Vila Real, Portugal; 3 Associate Laboratory for Animal and Veterinary Science (AL4AnimalS), 1300-477 Lisboa, Portugal; 4 Department of Chemistry and Biochemistry, Faculty of Sciences, LAQV, REQUIMTE, University of Porto, 4099-002 Porto, Portugal

**Keywords:** *Blastocystis* sp., cattle, *Cryptosporidium*, *Giardia duodenalis*, full length 18S, next generation sequencing, Oxford Nanopore Technologies

## Abstract

A total of 100 fecal samples collected from cattle in the district of Viana do Castelo, northern Portugal, were molecularly screened for *Cryptosporidium* spp., *Giardia duodenalis*, and *Blastocystis* sp. This study represents the first molecular survey of enteric protozoa in cattle from the district of Viana do Castelo, northern Portugal. *Blastocystis* sp. was the most frequently detected protozoan and exhibited considerable subtype diversity, including the zoonotic subtype ST3 and the potentially zoonotic subtypes ST10a and ST14, a circumstance highlighting the occurrence in cattle of *Blastocystis* subtypes with potential public health relevance and the need to further investigate their cross-host transmission dynamics. *Giardia duodenalis* was detected at a relatively low occurrence, although unsuccessful multilocus sequence typing (MLST) amplification prevented assessment of its assemblages and zoonotic potential. *Cryptosporidium* spp. were not detected, likely reflecting the predominance of adult cattle in the sampled population. Overall, these findings contribute to the understanding of the molecular epidemiology of enteric protozoa in Portuguese cattle and provide updated baseline geographical data for northern Portugal. Continued molecular surveillance using optimized genotyping approaches and larger, more representative sample sets will be essential to better characterize the diversity, epidemiology, and zoonotic importance of these parasites within a One Health framework.

## 1. Introduction

Diarrheal diseases are still among the top 10 leading causes of death according to the World Health Organization (WHO), especially among the most vulnerable populations. Among the most common protozoan parasites infecting humans are *Cryptosporidium* spp., *Giardia duodenalis* and *Blastocystis* sp., with the first two being of particular relevance as they are consistently highlighted among the WHO’s emerging infectious disease research priorities [1], namely *Cryptosporidium hominis*, *Cryptosporidium parvum* and *Giardia duodenalis*. In 2025, specific guidelines for both protozoan genera were published regarding drinking-water quality, sanitation and health, emphasizing their public health relevance [2,3].

Concerning the last one, *Blastocystis* sp. is responsible for the infection of an estimated 1 billion people worldwide, mostly by subtypes (ST) ST1–ST4, which account for ~90% of infections [4,5].

The detection of enteric protozoa in fecal samples has traditionally relied on coprological approaches, including direct microscopy, concentration techniques, specific staining procedures and immunofluorescence or antigen-detection assays, with the diagnostic approach varying according to the parasite investigated. Although these methods remain valuable for routine screening, their sensitivity may be affected by low parasite burdens, intermittent shedding and difficulties in distinguishing morphologically similar organisms. Molecular methods, particularly conventional and real-time PCR followed by sequencing, provide increased analytical sensitivity and specificity while enabling species-, genotype-, assemblage- and subtype-level characterization, thereby offering important information for assessing transmission patterns and zoonotic potential [6,7,8]. Previous studies have demonstrated the circulation of these enteric protozoa in Portuguese cattle. In northern Portugal, *Cryptosporidium* spp. and *Giardia duodenalis* were detected in both calves and adult cattle, with *Cryptosporidium parvum* and *G. duodenalis* assemblage E predominating, while zoonotically relevant *Giardia* assemblages A and B were also identified [9]. More recent molecular surveys conducted across Portugal have further documented *G. duodenalis*, *Cryptosporidium* spp. and considerable *Blastocystis* subtype diversity in domestic and wild hosts, including livestock, reinforcing the need for geographically resolved molecular surveillance of these parasites [10,11].

However, molecular epidemiological data on these enteric protozoa in Portuguese cattle remain limited, particularly in herds from northern Portugal managed under semi-intensive production systems. This lack of information on the species and subtypes circulating in cattle limits the assessment of their zoonotic potential and of the possible role of cattle in environmental contamination. Furthermore, most available evidence focuses on individual parasites, whereas the simultaneous investigation of multiple protozoan taxa may provide a more comprehensive understanding of their circulation at the cattle–human–environment interface, within a One Health framework.

The present study aimed to assess the occurrence and molecular diversity of *Cryptosporidium* spp., *G. duodenalis* and *Blastocystis* sp. in cattle from northern Portugal and to evaluate their potential epidemiological relevance in the context of semi-intensive production systems.

## 2. Material and Methods

### 2.1. Sampling

**Figure 1:**
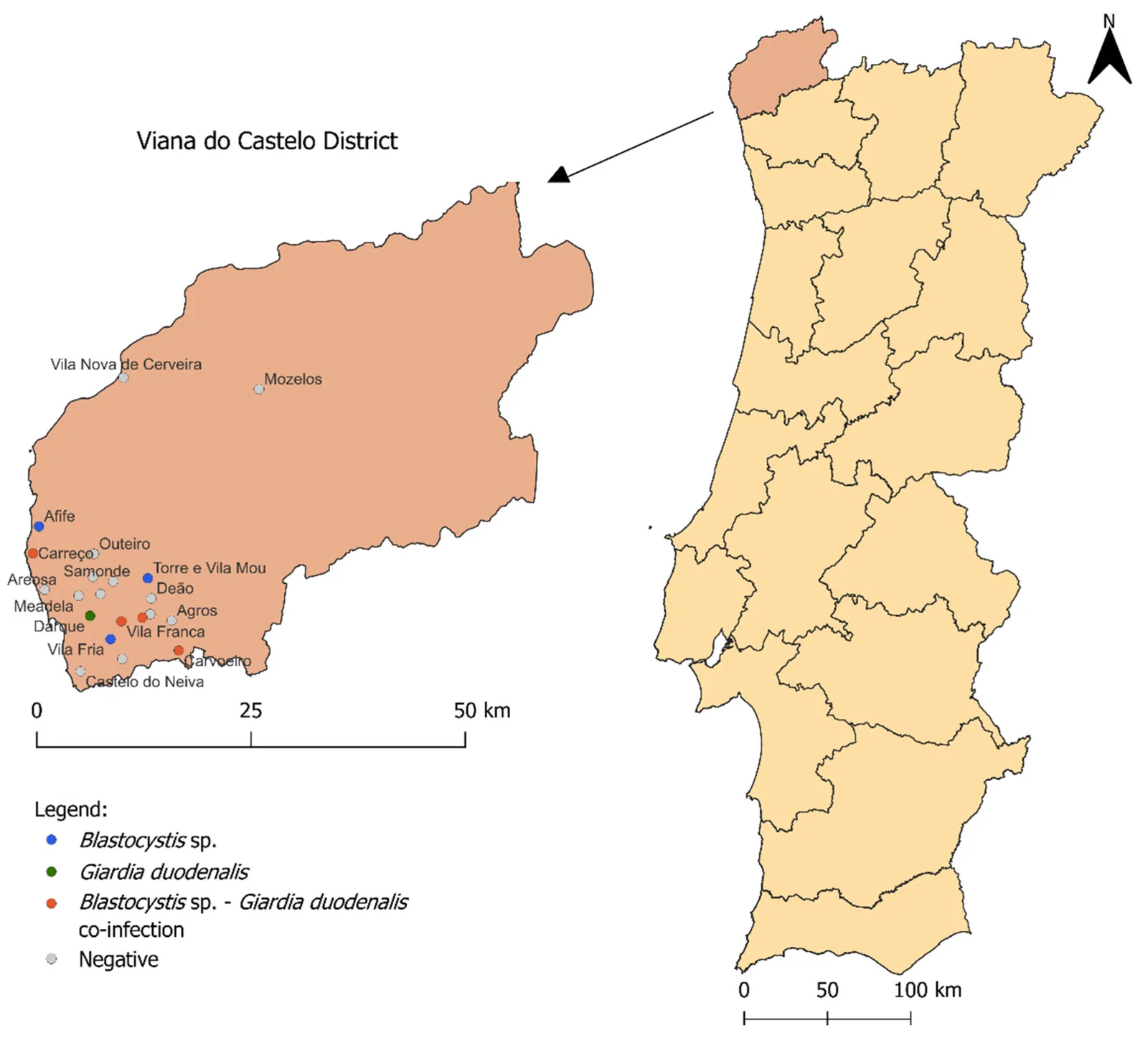
Geographic distribution of the cattle sampling sites in the district of Viana do Castelo, northern Portugal. The map on the right highlights the 21 farm locations of the district within mainland Portugal, and the enlarged map on the left shows the individual sampling locations included in this study.

A convenience sample of 100 fecal samples was collected between July and September 2024 from 21 cattle farms located across 21 parishes in the district of Viana do Castelo, northern Portugal. At the time of sampling, the district comprised 208 parishes, of which 21 (10.1%) were represented in the present study. The sampled population, raised under semi-intensive production conditions, comprised 78 adult cows, 5 heifers, and 17 calves. Of these, 17 adult cattle (21.8%) and 15 calves (88.2%) presented diarrhea. Samples were collected from the topmost layer of freshly deposited fecal material at the animals’ housing facilities, with sampling restricted to the surface layer to minimize the risk of contamination with environmental soil. Individual disposable gloves were used for each sample to prevent cross-contamination between animals, and samples were placed in sterile, labeled containers and stored at −20 °C until further processing. The total herd size of each sampled farm was not systematically recorded. The geographical distribution of sampling locations is shown in Figure 1, while detailed sampling data are provided in Supplementary Material Table S1.

### 2.2. DNA Extraction

DNA extraction was performed using the PurePrep96 magnetic-bead-based extraction system (MolGen^®^, Veenendaal, The Netherlands), according to manufacturer instructions. Before automated extraction, stool samples were prepared by diluting them in PBS to obtain a 10% suspension. The suspensions were vortexed thoroughly to ensure complete homogenization and then centrifuged at 8000× *g* for 5 min (Eppendorf, Hamburg, Germany) to pellet debris and concentrate particulate material. A total of 200 µL of the resulting supernatant was transferred to the PurePrep96 magnetic-bead-based extraction system, which automatically performed cell lysis, binding, and washing and provided a final elution of 100 µL of purified genomic DNA.

### 2.3. Molecular Detection of Protozoa

#### 2.3.1. *Cryptosporidium* spp.

*Cryptosporidium* spp. were identified using a nested PCR approach targeting the SSU rRNA gene. The primary reaction employed the outer primers CR-P1 (5′-CAGGGAGGTAGTGACAAGAA-3′) and CR-P2 (5′-TCAGCCTTGCGACCATACTC-3′), while the secondary reaction used the inner primers CR-P3 (5′-ATTGGAGGGCAAGTCTGGTG-3′) and CPB-DIAGR (5′-TAAGGTGCTGAAGGAGTAAGG-3′), as described by [12]. Both reactions followed the same thermal conditions: an initial denaturation step at 95 °C for 5 min, 40 amplification cycles (94 °C for 2 s, 50 °C for 5 s, and 72 °C for 5 s), and a final extension at 72 °C for 10 min. The primary reaction was performed in a final volume of 15 µL, comprising 7.5 µL of Speedy Supreme NZYTaq 2× Green Master Mix, 0.4 µM of each primer, and 2 µL of DNA template. The secondary reaction, in a final volume of 25 µL, contained 12.5 µL of Speedy Supreme NZYTaq 2× Green Master Mix, 0.4 µM of each primer, and 2 µL of DNA template.

#### 2.3.2. *Giardia duodenalis*

*Giardia duodenalis* detection was carried out by first performing and screening by qPCR, targeting a 62-bp fragment of the SSU-rRNA gene using the primers Gd-80F (5′-GACGGCTCAGGACAACGGTT-3′) and Gd127-R (5′-TTGCCAGCGGTGTCCG-3′) together with a FAM/BHQ1-labeled probe (5′-FAM–CCCGCGGCGGTCCCTGCTAG–BHQ1-3′). Reactions were performed in a final volume of 20 µL, containing 10 µL of Xpert Fast Probe 2× Mastermix (uni) (Grisp^®^, Porto, Portugal), 0.4 µM of each primer, 0.2 µM of probe, and 4 µL of DNA template. Amplification consisted of an initial denaturation step at 95 °C for 3 min, followed by 45 cycles of denaturation at 95 °C for 5 s and annealing at 60 °C for 20 s.

Samples testing positive by qPCR were subsequently characterized using a sequence-based multilocus genotyping (MLST) approach targeting three genes: triose phosphate isomerase (*tpi*), *β*-giardin (*bg*), and glutamate dehydrogenase (*gdh*). The *tpi* and *bg* loci were each amplified by separate nested PCRs, whereas *gdh* was amplified by semi-nested PCR.

For *tpi*, amplification used the outer primers AL3543 (5′-AAATIATGCCTGCTCGTCG-3′) and AL3546 (5′-CAAACCTTITCCGCAAACC-3′) in the primary reaction and the inner primers AL3544 (5′-CCCTTCATCGGIGGTAACTT-3′) and AL3545 (5′-GTGGCCACCACICCCGTGCC-3′) in the secondary reaction [13]. For *bg*, the primary reaction used the outer primers G7_F (5′-AAGCCCGACGACCTCACCCGCAGTGC-3′) and G759_R (5′-GAGGCCGCCCTGGATCTTCGAGACGAC-3′), while the secondary reaction used the inner primers G99_F (5′-GAACGAACGAGATCGAGGTCCG-3′) and G609_R (5′-CTCGACGAGCTTCGTGTT-3′) [14]. For *gdh*, the primary reaction used primers GDHeF (5′-TCAACGTYAAYCGYGGYTTCCGT-3′) and GDHiR (5′-GTTRTCCTTGCACATCTCC-3′), and the secondary reaction used the primers GDHiF (5′-CAGTACACCTCYGCTCTCGG-3′) and GDHiR [15].

All reactions shared the same thermal conditions, namely an initial denaturation step at 95 °C for 5 min, followed by 40 cycles of 94 °C for 2 s, locus-specific annealing for 5 s, and 72 °C for 5 s, with a final extension at 72 °C for 10 min. Annealing temperatures were 50 °C and 55 °C for both the primary and secondary reactions of *tpi* and *gdh*, respectively, whereas for *bg* the annealing temperature was 65 °C for the primary reaction and 55 °C for the secondary reaction.

#### 2.3.3. *Blastocystis* sp.

Molecular detection of *Blastocystis* sp. was carried out using real-time SYBR Green PCR (qPCR) as described by Poirier et al. [16]. The primer pair BL18SPPF1/BL18SR2PP, targeting the SSU rRNA gene, was used to amplify a fragment of approximately 300 bp. PCR reactions were performed on a CFX thermocycler (Bio-Rad, Hercules, CA, USA). Reaction mixtures were prepared using the Xpert Fast SYBR (Uni) Blue mix (GRiSP^®^, Porto, Portugal), following the manufacturer’s protocol. *Blastocystis*-positive samples with the expected melting curve were subjected to Sanger sequencing. Samples showing evidence of mixed infection, as indicated by multiple peaks in the Sanger sequencing chromatograms, were subsequently subjected to PCR amplification targeting the full-length 18S rRNA gene.

PCR targeting the full-length 18S rRNA gene was performed using the primer pair SSU-F1/SSU-R1 [17]. Reactions were prepared with a high-fidelity, long-template enzyme (Xpert AmpliFi Hotstart Mastermix, GRiSP^®^, Porto, Portugal) and run on a Bio-Rad thermocycler (Bio-Rad, Hercules, CA, USA). Cycling conditions consisted of an initial denaturation at 95 °C for 5 min, followed by 35 cycles of 94 °C for 2 s, 60 °C for 5 s, and 72 °C for 10 s, with a final extension at 72 °C for 10 min. Positive amplicons of full-length 18S sequences were submitted for ONT-Next Generation Sequencing to confirm the presence of mixed infections.

### 2.4. Sequencing

Nested and semi-nested PCR amplicons were separated on a 1% agarose gel run for 30 min at 120 V to confirm the expected size. Amplicons of the expected size, and of the expected melting temperature for *Blastocystis* qPCR, were purified using the Exo/SAP GoPCR Purification kit (GRiSP^®^, Porto, Portugal) and subsequently sequenced bidirectionally by Sanger sequencing. Sequences were edited, aligned, and analyzed in BioEdit Sequence Alignment Editor (v7.2.5), and the resulting consensus sequences were compared against the NCBI GenBank database using BLASTN 2.17.0+.

*Blastocystis* samples identified as mixed infections by Sanger sequencing, and subsequently confirmed positive by full-length 18S PCR, were sequenced using Oxford Nanopore Technology (ONT) on a PromethION 24 instrument (ONT, Oxford, UK) equipped with an R10.4.1 flow cell. Sequencing libraries were prepared with the Native Barcoding Kit 96 V14 (SQK-NBD114.96; ONT), following the manufacturer’s protocol. Raw reads were basecalled in super-accuracy mode using ont-dorado-for-promethion v7.4.12, applying a minimum Q-score threshold of 10, while adapter and barcode trimming were carried out within MinKNOW v26.01.21 (ONT).

### 2.5. Phylogenetic Analysis

For phylogenetic analysis, reference sequences were retrieved from the *Blastocystis* curated database (https://entamoeba.lshtm.ac.uk/ref.blasto.txt (accessed on 26 June 2026)) and aligned using MAFFT v7.490 with the L-INS-i algorithm to ensure high accuracy for diverse sequences [18]. Maximum Likelihood phylogenetic trees were constructed in IQ-TREE v3.0.1 [19] using 1000 bootstrap replicates and the K2P + I + G4 substitution model to ensure consistency with prior studies. The phylogenetic tree was rooted on the ST15/ST28 cluster, which represents an early-diverging lineage of *Blastocystis*. The resulting tree was further annotated and visually enhanced using the Interactive Tree of Life (iTOL) platform v7 [20].

### 2.6. Bioinformatic Analysis

A custom bioinformatic pipeline developed in-house was employed to process full-length 18S rRNA gene amplicon sequences generated using ONT sequencing. Raw sequence reads were initially assessed using FastQC (v2.8.0), followed by length and quality filtering with NanoFilt (minimum Phred quality score of 7, read length of 1600 to 1800 bp). Primer sequences were removed using Cutadapt (v.4.4) (maximum error rate of 0.2), retaining reads detected in either orientation. Putative *Blastocystis* reads were identified by BLASTn against a curated *Blastocystis* reference database (https://entamoeba.lshtm.ac.uk/ref.blasto.txt (accessed on 26 June 2026)), retaining only sequences with a minimum percentage identity and query coverage of 90%. Chimeric sequences were subsequently identified and removed using the reference-based “uchime_ref” algorithm implemented in VSEARCH (v.2.27.0), using the PR2 database as the reference to facilitate the detection of chimeras involving diverse eukaryotic taxa. The remaining sequences were clustered at 98% sequence identity using “cluster_fast” to generate representative centroid sequences. The corresponding reads were mapped to the representative centroids using minimap2, followed by one round of consensus polishing with Racon with default parameters. The polished centroids were subsequently re-clustered at the same identity threshold to consolidate redundant sequences before a final round of consensus refinement was performed with Medaka, yielding the final *Blastocystis* consensus sequences.

## 3. Results

Among the 100 stool samples analyzed in this study, *Cryptosporidium* spp. DNA was not detected. *Giardia duodenalis* was detected only by qPCR, with an occurrence of 6% (6/100, 95% CI: 2.8–12.5%), in 5 cows (3 without diarrhea and 2 with diarrhea) and one calf with diarrhea. No positive results were obtained by MLST, precluding genotype determination for this species.

**Figure 2:**
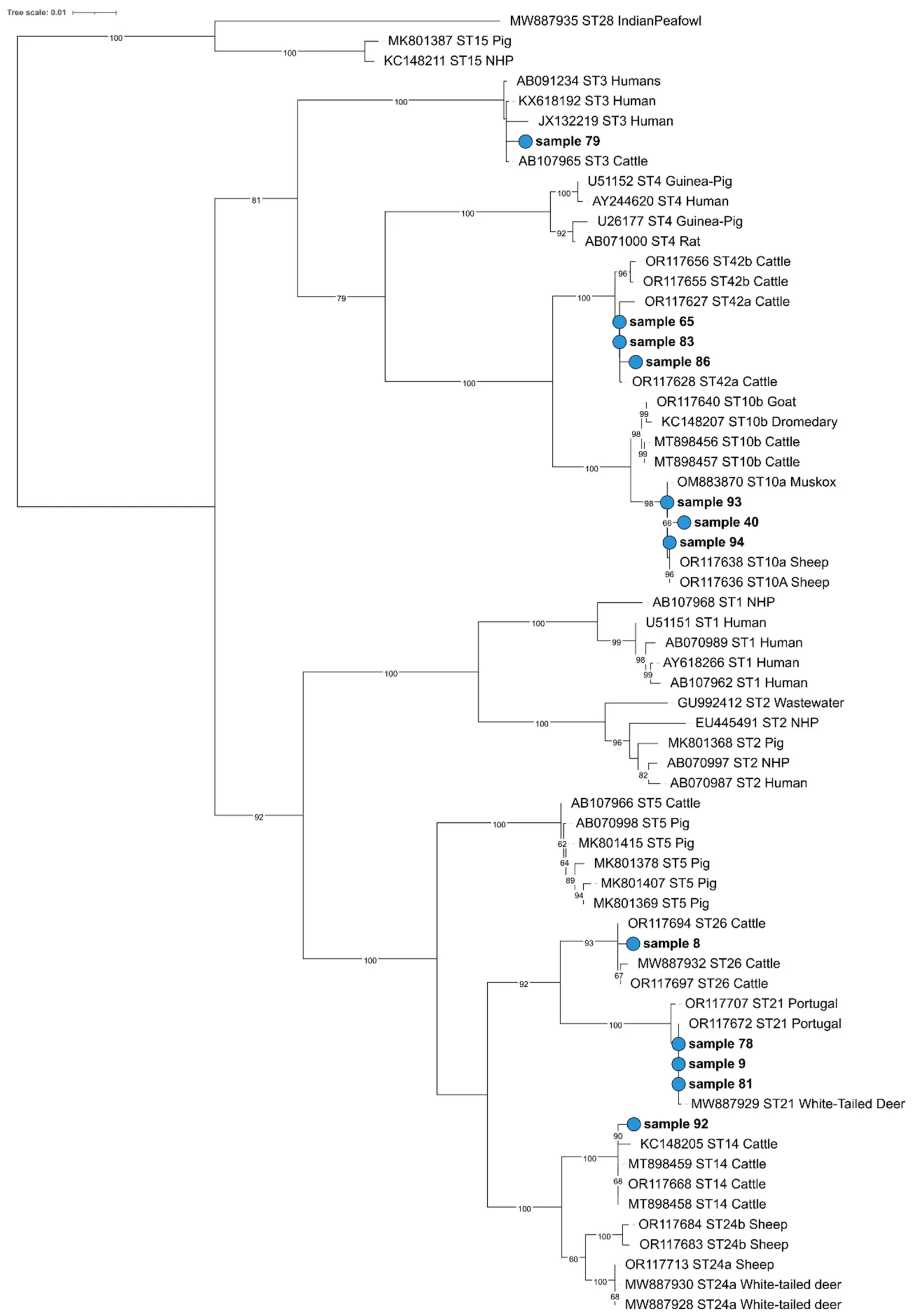
Phylogenetic tree of *Blastocystis* sp. subtypes (ST) constructed using full-length SSU rRNA gene sequences. Sequences generated by qPCR in this study are highlighted in bold and indicated by blue circles, whereas reference sequences are shown without formatting and are labelled with their respective subtypes and host of origin. The tree was inferred using IQ-TREE under the K2P + I + G4 substitution model with 1000 bootstrap replicates. Only bootstrap support values ≥ 60% are shown.

**Figure 3:**
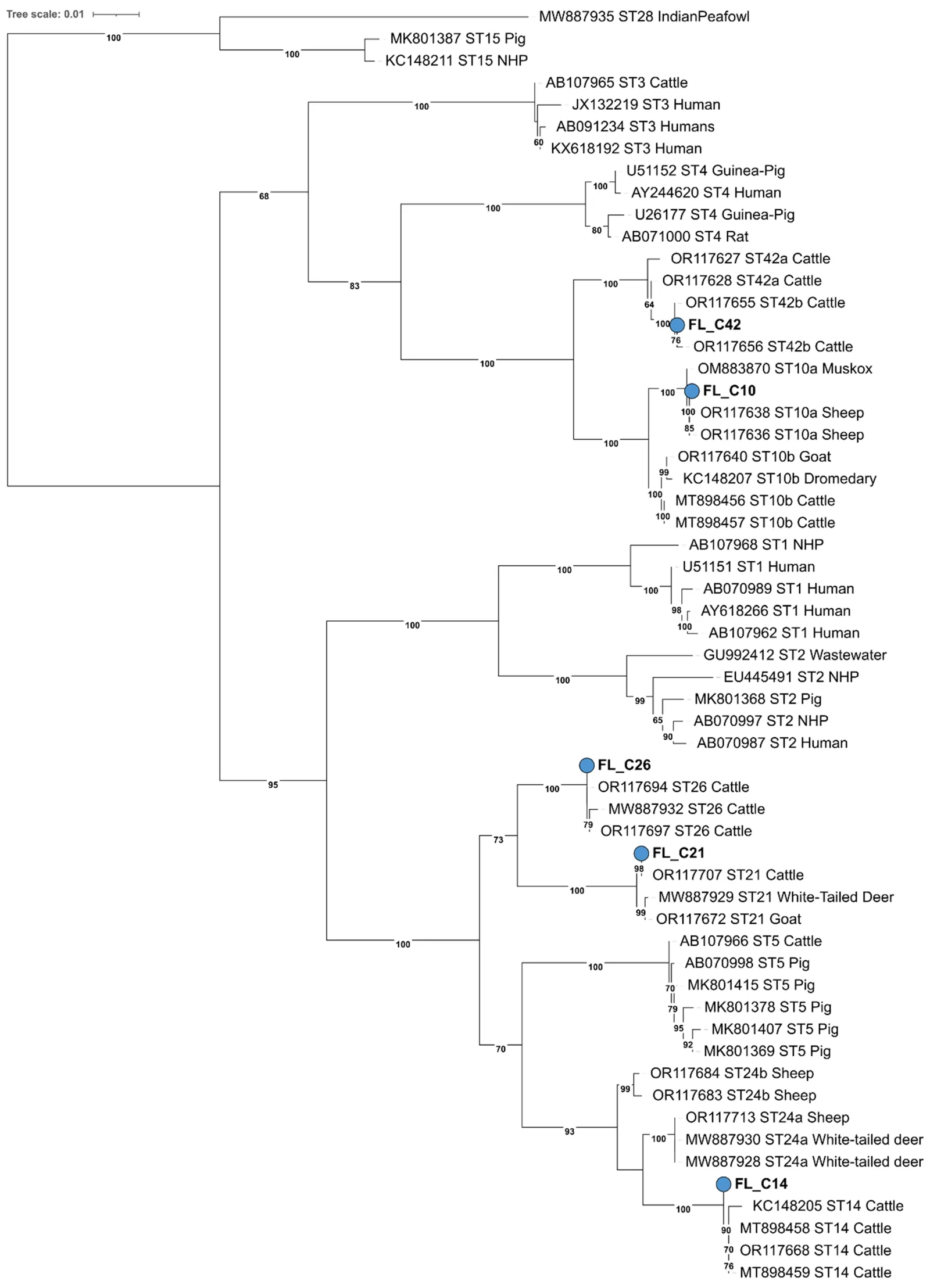
Phylogenetic tree of full length *Blastocystis* sp. subtypes (ST) constructed using full-length SSU rRNA gene sequences. Sequences generated by ONT in this study are highlighted in bold and indicated by blue circles, whereas reference sequences are shown without formatting and are labelled with their respective subtype and host of origin. The tree was inferred using IQ-TREE under the K2P + I + G4 substitution model with 1000 bootstrap replicates. Only bootstrap support values ≥ 60% are shown.

*Blastocystis* sp. was the most prevalent protozoan among cattle, with an occurrence of 20% (20/100, 95% CI: 13.3–28.9%) by qPCR. Twelve samples carried a single subtype, namely ST3 (*n* = 1), ST10a (*n* = 3), ST14 (*n* = 1), ST21 (*n* = 3), ST26 (*n* = 1), and ST42a (*n* = 3), as represented in Figure 2. The remaining eight samples showed potential mixed infection and were further studied for the full-length 18S rRNA gene; of these samples, one was confirmed as a mixed infection comprising five subtypes (ST10a, ST14, ST21, ST26, and ST42a) (Figure 3), while subtype assignment could not be achieved for the remaining seven samples. *Blastocystis*-positive samples were submitted to GenBank with accession numbers PZ712191-PZ712195 (near full-length 18S rRNA gene, ONT) and PZ712196- PZ712207 (partial 18S rRNA gene, Sanger sequencing).

Among the 20 *Blastocystis*-positive animals, 11 were adults, two were heifers, and seven were calves. Among adults, five animals carried a single subtype, comprising ST10a, ST14, ST21, ST26, and ST42a; five had an undetermined subtype; and one had a confirmed mixed infection comprising ST10a, ST14, ST21, ST26, and ST42a. Only one *Blastocystis*-positive adult presented diarrhea, and the subtype of this animal was undetermined. The adult with the confirmed mixed infection was non-diarrheic. Both ST42a-positive heifers were also non-diarrheic. Among calves, six of the seven *Blastocystis*-positive animals presented diarrhea, whereas one was non-diarrheic. Five calves carried a single subtype, comprising ST10a (*n* = 2), ST21 (*n* = 2), and ST3 (*n* = 1), while two had an undetermined subtype. The distribution of protozoan-positive and -negative samples according to animal age category is summarized in Table 1.

Of the six *G. duodenalis*-positive animals, three were co-infected with *Blastocystis* sp.: two non-diarrheic adults, carrying ST10a and an undetermined subtype, respectively, and one diarrheic calf carrying ST21. The remaining three *G. duodenalis*-positive animals were negative for *Blastocystis* sp. Individual-level data, including farm of origin, animal category, diarrheal status, *Blastocystis* subtype, and *G. duodenalis* detection, are provided in Supplementary Table S1.

## 4. Discussion

The present study revealed the presence of enteric protozoa in cattle from the Viana do Castelo district, northern Portugal. *Blastocystis* sp. was the most frequently detected protozoan, with an occurrence of 20% and considerable subtype diversity, including ST3, ST10a, ST14, ST21, ST26, and ST42a. Despite their unclear pathogenicity, some subtypes, namely ST1-ST8, have been suggested to cause disease [21,22,23,24,25,26,27], and some have also been associated with zoonotic potential, namely ST1–ST10, ST12–ST14, ST16, ST23, ST35, and ST41 [28,29].

In Portugal, cattle production has placed the country among the top 15 largest cattle producers in the European Union according to Eurostat data, with approximately 1.47 million heads, underscoring the sector’s economic importance [30]. In the North region specifically, cattle farming is closely associated with small to medium-sized herds managed under semi-intensive or extensive systems [31], with direct exposure to pasture, soil, and surface water. This close interface between cattle, farm workers, and the environment has direct implications for the dissemination of protozoa. *Cryptosporidium parvum* oocysts, for example, can be shed at concentrations exceeding 10^7^ per gram of feces [32,33], creating a risk of surface contamination and also groundwater following manure application or runoff, and *Giardia* cysts are similarly environmentally robust [34,35]. *Blastocystis* sp. follows a similar pattern; i.e., subtypes ST10 and ST14 are most frequently identified in cattle, with shared subtypes between animals and humans indicating potential zoonotic transmission, and nearness to livestock environments has been associated with increased risk of human infection [28,36]. Most of these subtypes are predominantly associated with cattle, whereas ST3 is among the most frequently reported in humans, highlighting the potential role of cattle as reservoirs of zoonotic *Blastocystis* subtypes.

Of particular interest, ST3 was detected in a single animal. Although this subtype is frequently reported in humans, it has also been identified in cattle and other non-human hosts, and its detection alone does not allow the direction of transmission to be established. Previous investigations involving cattle and in-contact humans have suggested that the occurrence of ST3 at a low frequency in cattle may result from occasional exposure to human or other animal fecal material, either directly or indirectly through contaminated water or the farm environment [37,38]. Conversely, the possibility of cattle contributing to cross-host transmission cannot be excluded. In the present study, no samples or parasitological information from animal handlers was available, preventing assessment of whether the ST3-positive animal could have acquired the infection from humans or represented a potential source of human exposure. Future studies combining paired sampling of cattle, animal handlers, and environmental matrices, together with higher-resolution molecular characterization, would therefore be required to clarify the transmission direction and epidemiological significance of ST3 in this setting.

Although primarily associated with ruminants, particularly cattle, ST10a and ST14 have also been reported in human infections. Despite the uncertain pathogenicity of *Blastocystis* sp., several studies have associated particular subtypes with gastrointestinal disease, although this relationship remains controversial and requires further investigation [39,40]. Nine samples were presumptively identified as mixed *Blastocystis* infections by qPCR. However, only one was confirmed by ONT sequencing, while the remaining positive samples could not be assigned to a specific subtype. This discrepancy is likely attributable to the higher analytical sensitivity of qPCR and the reduced amplification efficiency of low-abundance templates during amplicon sequencing, despite the greater sequencing depth provided by ONT [41]. Nevertheless, the confirmed mixed infection, comprising ST10a, ST14, ST21, ST26, and ST42a, demonstrates that cattle can simultaneously harbour multiple *Blastocystis* subtypes, consistent with previous reports [38]. In Portugal, *Blastocystis* occurrence has been reported at 32.2% in cattle from the mainland [38] and 14.7% in cattle from Terceira Island (Azores) [42], compared with the 20% observed in the present study. Across Europe, reported prevalences also vary considerably, ranging from 8% in Italy to 34.3% in Spain and 54.8% in France [43,44,45]. Such variations are likely influenced by differences in animal age, husbandry practices, geographical location, sample size, and molecular detection methods. Despite these differences, *Blastocystis* remained the most frequently detected protozoan in the present study, supporting its widespread occurrence in cattle populations.

Phylogenetic analysis further enabled the detected *Blastocystis* subtypes to be contextualized relative to sequences previously reported from different hosts and geographical regions, providing insight into their broader circulation and genetic relatedness. However, the clustering observed should not be interpreted as evidence of a specific geographical source or direct transmission pathway, as the SSU rRNA marker and the currently available reference sequences do not provide sufficient resolution for such inference.

The phylogenetic placement of some sequences within subtype lineages that also contained isolates previously obtained from wild ruminants, including muskox and white-tailed deer, further highlights the broad host range of some *Blastocystis* subtypes. This observation raises the possibility that wildlife may contribute to the maintenance and circulation of these subtypes at the livestock–wildlife–environment interface, particularly where cattle and wild animals share pasture, water sources, or other environmentally contaminated resources. However, phylogenetic relatedness alone cannot demonstrate direct wildlife-to-cattle transmission, and no wild animals were sampled in the present study. Therefore, the source and direction of transmission remain unresolved and would require simultaneous molecular surveillance of livestock, sympatric wildlife, and environmental matrices.

The second most frequently detected protozoan was *G. duodenalis*, with an occurrence of 6% determined by qPCR targeting the SSU rRNA gene. However, molecular characterization to the subassemblage level was unsuccessful, as none of the three MLST loci (*tpi*, *gdh*, and *bg*) could be amplified. Similar difficulties have previously been reported in studies conducted in Portugal and Spain [10,46]. In the present study, Ct values of *G. duodenalis*-positive samples ranged from 28 to 37, suggesting that the low concentration of target DNA may have hindered successful amplification of the MLST loci. Consequently, the zoonotic potential of the detected *G. duodenalis* isolates could not be assessed, as reliable evaluation requires characterization at both the assemblage and subassemblage levels. Assemblages A and B are considered the principal zoonotic assemblages and are shared between humans and animals, whereas the remaining assemblages (C to H) are predominantly host-adapted, with assemblage E being the genotype most commonly detected in cattle [47]. Without molecular characterization, it remains unknown whether the *G. duodenalis* strains circulating in this cattle population belong to the zoonotic assemblages A or B or to the cattle-adapted assemblage E. Within Portugal, *G. duodenalis* prevalences of 44.4% in Terceira Island and 14.9% and 14.1% in mainland cattle have previously been reported [9,10,48]. In these studies, assemblage E was the predominant genotype, whereas the zoonotic assemblages A and B were detected only occasionally. The lower occurrence observed in the present study may reflect differences in animal age, management practices, geographical location, sample size, or methodological approaches. Similarly, the prevalence detected here was lower than those reported in several other European countries, including Poland (10.9%) [49], Spain (12.4%) [50], and Italy (32%) [51].

Finally, although *Cryptosporidium* spp. were not detected in the present study, they remain among the most important enteric protozoa affecting cattle worldwide due to their impact on animal health and their zoonotic potential. Several species of *Cryptosporidium* have been reported in cattle, with *C. parvum* being the most relevant from a public health perspective because of its ability to infect humans [52], whereas species such as *Cryptosporidium bovis*, *Cryptosporidium ryanae*, and *Cryptosporidium andersoni* are considered host-adapted [53]. The absence of *Cryptosporidium* spp. in the present study aligns with previous reports from Portugal [10,54]; however, an occurrence of 42.3%% has been described in calves from Terceira Island [48]. Several factors may explain the absence of *Cryptosporidium* spp. in the present study. Age is one of the main determinants of infection, with the highest prevalence generally reported in pre-weaned calves, while infections become progressively less frequent in older animals owing to the development of partial immunity. As the sampled population consisted predominantly of adult cattle, the likelihood of detecting *Cryptosporidium* could be expected to be lower. In addition, differences in farm management, environmental conditions, sampling strategy, and diagnostic methods may also contribute to variations in occurrence among studies. Although *Cryptosporidium* spp. were not detected, continued surveillance remains important given the parasite’s veterinary importance and the zoonotic risk posed by species such as *C. parvum*.

This study has several limitations that should be considered when interpreting the results. Firstly, the sampling was restricted to 100 cattle from a district in northern Portugal and was conducted over a relatively short period, which may limit the generalizability of the findings to other geographical regions or production systems. Moreover, the spatial distribution of the sampled farms was uneven, with most locations concentrated in the coastal and southern areas of the Viana do Castelo district. As the study relied on convenience sampling rather than a geographically stratified design, the findings should therefore not be considered fully representative of the entire district or, more broadly, of northern Portugal.

Secondly, the sampled population consisted predominantly of adult animals, whereas enteric protozoa such as *Cryptosporidium* spp. and *Giardia duodenalis* are generally more prevalent in young calves. Consequently, the absence of *Cryptosporidium* spp. and the relatively low occurrence of *G. duodenalis* may not accurately reflect the epidemiological situation in younger age groups. Additionally, because fecal samples were collected from freshly voided material rather than directly from the rectum, the possibility of environmental contamination cannot be completely excluded, although sampling was restricted to the uppermost portion of freshly deposited feces to minimize this risk. Furthermore, the inability to amplify the MLST loci of *G. duodenalis* prevented assemblage characterization and, therefore, assessment of the zoonotic potential of the detected isolates. Likewise, although several *Blastocystis* sp. samples were suggestive of mixed infections, only one could be confirmed by ONT sequencing, likely owing to the low abundance of target DNA. Future studies should include larger and more geographically representative sample collections encompassing different production systems and age groups, together with optimized molecular approaches to improve genotyping success and the detection of mixed infections.

## 5. Conclusions

This study provides a molecular survey of enteric protozoa in cattle from the Viana do Castelo district, northern Portugal. *Blastocystis* sp. was the most frequently detected protozoan and exhibited considerable subtype diversity, including the zoonotic subtype ST3 and the potentially zoonotic subtypes ST10a and ST14, highlighting the potential role of cattle as reservoirs of *Blastocystis* strains of public health relevance. *Giardia duodenalis* was detected at a relatively low occurrence, although unsuccessful MLST amplification prevented assessment of its assemblages and zoonotic potential. *Cryptosporidium* spp. were not detected, likely reflecting the predominance of adult cattle in the sampled population. Overall, these findings contribute to the understanding of the molecular epidemiology of enteric protozoa in Portuguese cattle and provide updated baseline data for northern Portugal. Continued molecular surveillance using optimized genotyping approaches and larger, more representative sample sets will be essential to better characterize the diversity, epidemiology, and zoonotic significance of these parasites within a One Health framework.

## Figures and Tables

**Table 1:** Molecular detection of enteric protozoa according to cattle age category. Results are presented as the number and percentage of positive and negative samples for *Cryptosporidium* spp., *Giardia duodenalis*, and *Blastocystis* sp.

Age Category	*n* Tested	*Cryptosporidium* Positive *n* (%)	*Cryptosporidium* Negative *n* (%)	*Giardia* Positive *n* (%)	*Giardia* Negative *n* (%)	*Blastocystis* Positive *n* (%)	*Blastocystis* Negative *n* (%)
Adults	78	0 (0.0)	78 (100.0)	5 (6.4)	73 (93.6)	11 (14.1)	67 (85.9)
Heifers	5	0 (0.0)	5 (100.0)	0 (0.0)	5 (100.0)	2 (40.0)	3 (60.0)
Calves	17	0 (0.0)	17 (100.0)	1 (5.9)	16 (94.1)	7 (41.2)	10 (58.8)
Total	100	0 (0.0)	100 (100.0)	6 (6.0)	94 (94.0)	20 (20.0)	80 (80.0)

## Data Availability

*Blastocystis*-positive samples were submitted to GenBank with accession numbers PZ712191-PZ712195 (near full-length 18S rRNA gene, ONT) and PZ712196-PZ712207 (partial 18S rRNA gene, Sanger sequencing).

## Supplementary Materials

The following supporting information can be downloaded at: https://www.mdpi.com/article/10.3390/pathogens15090937/s1, Table S1: Individual characteristics and molecular findings of cattle positive for *Blastocystis* sp. and/or *Giardia duodenalis*.
