# Supplementary material for: Molecular Detection of Enteric Protozoa in Cattle from North Portugal
Source: Pathogens. 2026 Sep 4;15(9):937. doi: 10.3390/pathogens15090937 (PMC13610738; doi:10.3390/pathogens15090937)
Supplement: Supplementary file 1 [file pathogens-15-00937-s001.zip › pathogens-4480407-supplementary-done.pdf]

pathogens

MDPI

Supplementary Materials

Table S1. Individual characteristics and molecular findings of cattle positive for *Blastocystis* sp. and/or *Giardia duodenalis*.

Sample ID	Farm origin / parish	Category	Diarrhea	*Blastocystis* sp.	*G. duodenalis*
4	Carreço	Adult	Yes	Negative	Positive
6	Carreço	Adult	No	Undetermined	Negative
8	Carreço	Adult	No	ST26	Negative
9	Carreço	Adult	No	ST21	Negative
34	Subportela	Adult	No	Negative	Positive
37	Darque	Adult	Yes	Negative	Positive
40	Vila Franca	Adult	No	ST10a	Positive
41	Vila Franca	Calf	No	Undetermined	Negative
43	Afife	Adult*	Yes	Undetermined	Negative
59	Vila Fria	Adult	No	Mixed (ST10a, ST14, ST21, ST26, ST42a)	Negative
65	Torre e Vila Mou	Heifer	No	ST42a	Negative
73	Cravoeiro	Adult	No	Undetermined	Negative
74	Carvoeiro	Adult	No	Undetermined	Negative
77	Carvoeiro	Calf	Yes	Undetermined	Negative
78	Carvoeiro	Calf	Yes	ST21	Negative
79	Carvoeiro	Calf	Yes	ST3	Negative
81	Carvoeiro	Calf	Yes	ST21	Positive
82	Carvoeiro	Adult	No	Undetermined	Positive
83	Carvoeiro	Heifer	No	ST42a	Negative
86	Subportela	Adult	No	ST42a	Negative
92	Vila Franca	Adult	No	ST14	Negative
93	Vila Franca	Calf	Yes	ST10a	Negative
94	Vila Franca	Calf	Yes	ST10a	Negative
